# Compressor Flow Perception via Deep Learning Modeling with Multi-Source Dynamic Fusion of Temporal Features by Bio-Inspired Optimization

**DOI:** 10.3390/biomimetics11070452

**Published:** 2026-06-30

**Authors:** Mingming Zhang, Yuying Zhao, Huan Li, Xi Nan, Ning Ma, Ruoyang Liu, Quan Wen

**Affiliations:** 1 School of Mathematics Statistics and Mechanics, Beijing University of Technology, Beijing 100124, China; bzhaoyuying@163.com; 2 Aero Engine Academy of China, Beijing 101304, China; nanxi_ut@outlook.com (X.N.); maning216@163.com (N.M.); ruoyang_liu@foxmail.com (R.L.); toutao40@163.com (Q.W.)

**Keywords:** aerodynamic instability, dispersion entropy, long short-term memory network, dung beetle optimizer, dynamic cross-attention

## Abstract

This is of significant engineering importance for enhancing the operation stability and reliability of aeroengines. To ensure the precise identification of aerodynamic instability, it proposes a deep learning model for multi-source fusion based on cross-attention and bidirectional Long Short-Term Memory (CA_BiLSTM) network. From a high-speed multistage compressor, multi-dimensional feature extraction is performed in the time domain, frequency domain, and entropy value range. Based on dispersion entropy, feature cross-identification is constructed with a multi-level early warning method. In response to the nonlinear aerodynamic parameters, Variational Mode Decomposition (VMD) and Dung Beetle Optimizer (DBO) for global optimization are integrated to construct a VMD_DBO_LSTM-coupled prediction model for aerodynamic stability. To address the limitation of single-point detection, this paper proposes a dual-channel fusion model based on cross-attention mechanism. Through shared convolution and dynamic weighting mechanism, the CA_BiLSTM model can precisely characterize the nonlinear features of the complex flow. It can fully integrate the complementary information of inlet and outlet signals, achieving the collaborative signal characterization. Its anti-interference capability is significantly superior to that of the original single-point signal. Combined with the dispersion entropy threshold, it can detect instability 1580 r in advance, effectively overcoming the problems of information deficiency and incomplete representation caused by traditional single-point monitoring.

## 1. Introduction

The development of modern aviation technology has placed higher demands on the safety and reliability. Relevant statistics show that accidents caused by engine failure account for over 41% of flight accidents, making it one of the main factors threatening aviation safety [1]. As the core component of an aeroengine, the stable operation of the compressor directly affects overall engine performance and flight safety. Aerodynamic instability phenomena such as rotating stall and surge are key triggers of engine failure. Such instabilities can cause severe flow separation, pressure pulsation, and blade fatigue. Under complex conditions, insidious secondary instability may be induced, further increasing the risk of engine damage [2]. Precise instability detection is strongly needed to provide support for active control [3].

The compressor of an aeroengine is characterized by strong nonlinearity, strong transience, and strong coupling, making the mechanism of flow instability extremely complex [4]. Compressor stall and surge mostly occur under high-pressure ratio and heavy-load conditions, triggering airflow oscillation and passage blockage, seriously endangering flight safety [5]. Affected by characteristics such as sudden evolution and weak precursor, traditional monitoring methods struggle to effectively capture early abnormal signals [6], increasing the difficulty of instability early warning. How to achieve precise identification of compressor stall, surge, and hidden instability states has become a core problem to be tackled in the field of aeroengine health management.

In the signal processing of feature extraction, common methods mainly include time-domain analysis, frequency-domain analysis, and time-frequency domain analysis. Time-domain analysis reflects signal dynamic characteristics by extracting statistical features such as mean, variance, and kurtosis. However, such methods are susceptible to noise interference and have poor adaptability to unstable operating conditions with low feature discrimination between different states [7,8]. Frequency-domain analysis, centered on Fourier transform and power spectrum analysis, can effectively identify frequency components, which is applicable to stable signals. It cannot capture transient anomaly information and is prone to spectrum aliasing under multiple coupling conditions, leading to a reduced diagnostic reliability [9,10]. Time-frequency analysis can reflect the time and frequency distribution characteristics of signals simultaneously. Among them, empirical mode decomposition and Hilbert-Huang transform can adaptively process nonlinear and unstable signals but are susceptible to mode mixing and endpoint effects. Wavelet transform possesses the capability of multi-resolution but suffers from fixed basis functions and insufficient matching with actual fault characteristics [11,12,13]. Therefore, traditional signal processing methods have significant limitations when dealing with strong noise and unstable and multi-fault coupled complex signals, making it difficult to meet the requirements of compressor instability identification under complex operating conditions.

In the field of fault diagnosis, entropy-based methods are used due to the excellent ability to quantify the complexity of nonlinear signals. Approximate entropy characterizes the irregularity of dynamic pressure signals, enabling early capture of weak nonlinear disturbances before instability, becoming an important means for early stall warning [14]. Sample entropy, as an improved algorithm over approximate entropy, effectively reduces dependence on data length. Combined with spatial modal analysis, it significantly improves the reliability and robustness of single-probe diagnosis [15]. Fuzzy entropy optimizes similarity measurement through fuzzy functions, is insensitive to parameter selection, and is suitable for identifying precursor features under different stall types such as spike type and modal wave [16]. Wavelet singular spectral entropy further integrates wavelet transform and singular value decomposition to achieve joint time-frequency domain feature extraction, offering a more sensitive warning effect [17]. However, most existing entropy methods suffer from problems such as insufficient utilization of amplitude information, insensitivity to subtle signal fluctuations, and limited noise immunity, making it difficult to accurately characterize the temporal complexity and dynamic mutation features during stall evolution.

Meanwhile, relying solely on signal feature analysis is still insufficient to fully reveal the intrinsic physical mechanisms of compressor instability. Traditional modeling methods also have limitations in practical early warning. The physical models such as the Moore–Greitzer model [18,19] often adopt simplified flow assumptions, making it difficult to reflect the complex internal local flow structures and unsteady disturbance in compressor. Although the extended nonlinear M-G model introduces rotor dynamics and rotating stall modes, it is still constrained by idealized conditions [20]. The two-dimensional incompressible rotating disturbance model developed based on the M-G model can describe the impact of rotating disturbance but cannot accurately capture weak precursors and nonlinear evolution processes [21], failing to meet the requirements of high precision and high robustness for engineering early warning.

With the rapid development of artificial intelligence, machine learning and deep learning methods have been widely applied in the field of mechanical fault assessment. Zhu proposed a fusion model of LSTM, fuzzy comprehensive evaluation, and analytic hierarchy process for wind turbines [22], achieving precise assessment of equipment operating status. In response to the need for compressor instability early warning, Hipple validated an LSTM model [23], and results showed that the regression scheme could predict stall occurrence 5–20 ms in advance. A CNN-BiLSTM hybrid model [24] was proposed to achieve high-precision prediction under multiple conditions. The BP neural network was integrated with fuzzy logic to reduce false alarms through temporal decision-making [25]. Zhang proposed a digital twin graph convolutional spatiotemporal fusion model, combining topological analysis and TCN-LSTM to improve prediction accuracy and early warning capability [26]. These studies indicate that AI methods can effectively mine instability evolution features, providing reliable theoretical and technical foundations for monitoring compressor aerodynamic instability. However, single-monitoring prediction models rely only on information from the single-location measurement. This makes it susceptible to local noise and flow interference, resulting in a poor robustness.

As a typical representative of biomimetics-inspired intelligent computing, bio-inspired metaheuristic optimization algorithms construct efficient global search frameworks by simulating biological foraging behaviors, population evolution, and ecological survival strategies in nature. Compared with traditional gradient-driven optimization methods, biomimetic optimization algorithms feature strong robustness and low requirements for problem convexity and differentiability. The excellent adaptive search capability has emerged as an indispensable technical solution for parameter tuning of signal processing algorithms and deep learning models applied to mechanical fault diagnosis and condition forecasting. For instance, the whale optimization algorithm, a biomimetic optimizer, has been introduced to optimize the LSTM network for axial compressor stall prediction, effectively improving the early warning performance [27].

As an emerging biomimetic algorithm, the Dung Beetle Optimizer (DBO) achieves a more effective balance between global exploration and local exploitation by mimicking the staged foraging, reproduction, and intraspecific competition behaviors of dung beetle populations. Its superior optimization performance has been validated in cross-domain engineering applications. It was adopted to adaptively optimize variational mode decomposition parameters for gearbox fault diagnosis, significantly enhancing the decomposition quality and feature extraction capability [28]. It was employed to tune the key hyperparameters of Transformer networks for urban traffic level of service prediction, remarkably improving the model prediction accuracy and generalization performance [29]. Motivated by the outstanding performance of biomimetic algorithms in addressing complex engineering parameter optimization problems, this paper introduces the DBO algorithm to realize adaptive global optimization of key parameters, so as to further improve the signal decomposition quality and the accuracy of aerodynamic instability prediction.

In summary, existing signal processing methods have insufficient adaptability to strong noise and unstable, and multi-sensor coupled data, making it difficult to accurately capture the weak nonlinear evolution of stall precursors. Although entropy-based methods can characterize signal complexity, the amplitude information is not fully utilized, and the response to transient mutations is lagged behind. Furthermore, traditional physical models are constrained by idealized assumptions, making it difficult to reflect the unsteady disturbances and local complex flow.

To address these limitations, this paper takes “Identification-Prediction-Fusion” as the main thread, proposing the following innovations:

• Instability classification via discrimination strategy. Dispersion entropy combined with a sliding window mechanism is introduced to quantitatively characterize signal complexity during stall evolution. Based on the three-sigma criterion, progressive warning thresholds are set, dividing the stall process into “Stable Stage-Precursor Stage-Stall Stage”, achieving refined hierarchical warning.
• Transient modeling with VMD optimized by DBO. To address the problem of VMD parameters relying on manual experience, DBO is introduced to adaptively optimize the number of modes and penalty factor with minimum envelope entropy. An LSTM architecture of “Parameter Optimization-Data Decomposition-Component Layered Training-Result Fusion” is constructed, effectively enhancing the representation capability of complex time series signals.
• Multi-source fusion deep learning model integrating cross-attention mechanism and bidirectional LSTM. Targeting the spatiotemporal differences in compressor inlet and outlet sensors, the CA_BiLSTM model is designed. It extracts local and long-term dependency features through shared convolution and BiLSTM and utilizes cross-attention to achieve dynamic weight adaptive allocation. It introduces the physical constraint and residual optimization module, enhancing the robustness of stall recognition under multi-source information fusion.

The structure of this paper is as follows: First, focus on the signal acquisition and feature extraction of high-speed compressor, including filtering and noise reduction, resampling strategy, and multi-domain time-frequency analysis. Then, combined with dispersion entropy, classification criterion for flow instability is constructed. Based on multi-dimensional feature cross-validation, refined classification of the stall evolution is achieved. Next, an aerodynamic stability model is proposed based on hybrid LSTM with a single monitor, combined with the DBO to optimize key parameters of VMD. Through multiple comparative experiments, the prediction accuracy and perception capability of the model are comprehensively evaluated. Finally, a CA_BiLSTM fusion model with multi-source is proposed. The fusion effectiveness is systematically validated from multiple dimensions, including dynamic weight allocation, anti-noise interference capability, and signal quality improvement.

## 2. Flow Stability Graded Discrimination Based on Entropy Features

### 2.1. Data Sources

**Figure 1:**
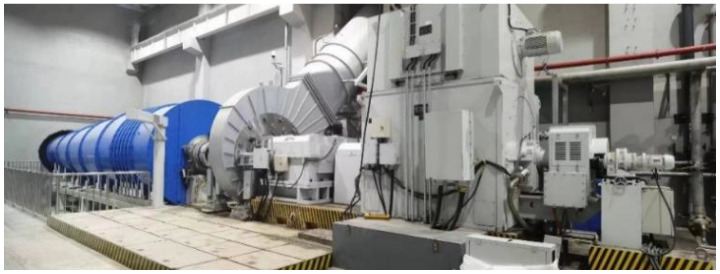
High-speed multistage axial compressor test rig.

The research object of this paper is a high-speed axial multistage compressor (Figure 1). And the rig specifications are indicated in Table 1.

**Figure 2:**
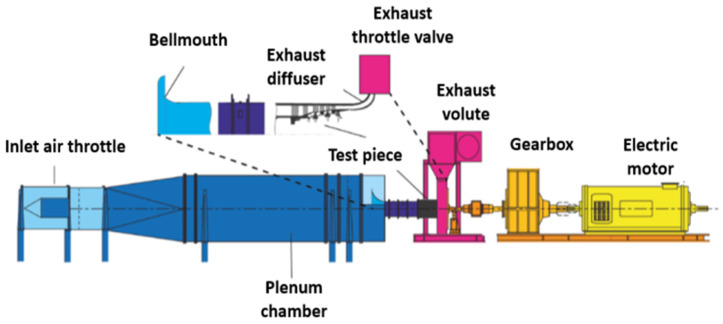
Multistage axial compressor principle.

**Figure 3:**
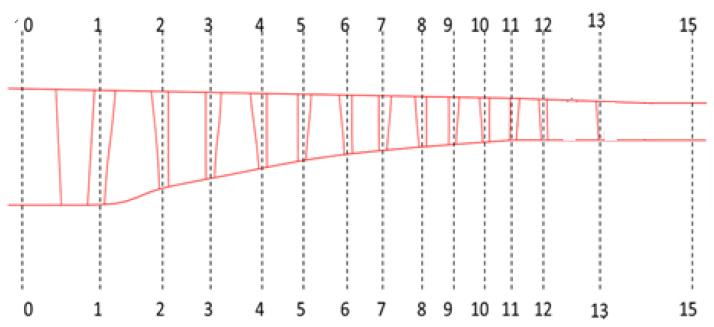
Measurement section schematic diagram.

Based on this multistage compressor platform (Figure 2), the flow dynamic pressure data of inlet and outlet sections in the compressor were completely recorded at a sampling frequency of 20,000 Hz, under an operating speed of 11,780 rpm, from a stable state to the occurrence of rotating stall. The measurement section distribution of the test rig is shown in Figure 3, with measurements located on the casing wall at Sections 0 and 15. This data acquisition provides continuous and reliable measured data support for subsequent work on rotating stall feature extraction, instability mechanism analysis, and identification model construction.

### 2.2. Data Preprocessing

**Figure 4:**
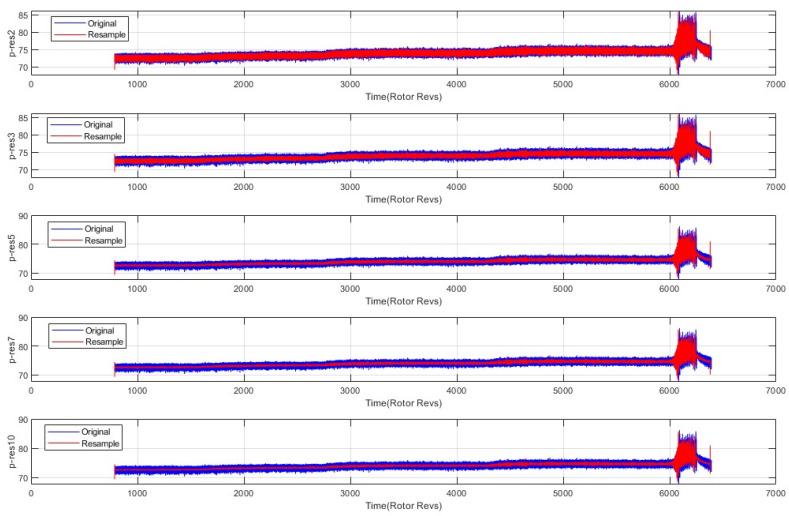
Comparisons of resampling at different ratios.

The data collected from experiments inevitably contains high-frequency redundant noise and acoustic disturbances. To improve the signal-to-noise ratio and avoid signal error caused by spectral aliasing during subsequent resampling, a Butterworth filter for high frequency filtering is used for the raw signal, setting the cutoff frequency to 1000 Hz. In axial compressors, the fundamental frequency of rotating stall is typically a fraction of the rotor rotational frequency—commonly ranging from approximately 25% to 50% of rotor speed. For the present test condition at 11,780 rpm (approximately 196.3 Hz rotational frequency), the stall cell propagation frequency is expected to lie well below 200 Hz. The 1000 Hz cutoff therefore preserves the stall fundamental frequency and its primary harmonics while effectively attenuating high-frequency acoustic noise and electrical interference. To raise the computational efficiency of input and output for the sampled data, resampling is performed after filtering, as shown in Figure 4.

By comparing resampling frequencies at multiples of 2, 3, 5, 7, and 10 times, data dimensionality reduction is achieved while preserving core characteristic information. As seen from Figure 4, the resampling of 5 times reduces the data dimension to one-fifth of the original, effectively filtering out most high frequency noise while preserving the key dynamic characteristics from stable flow to structure destabilization during the stall process. Reasonable data reduction allows subsequent feature extraction and model training to focus on the key characteristics of flow stall, thereby improving the accuracy of instability identification, enhancing generalization capability, and suppressing overfitting risks.

Based on the 5-time (4000 Hz) resampling process, in order to mitigate the interference of differing data magnitude levels on prediction results, this paper applies the function to smooth the raw data. Then min-max normalization is performed on the smoothed data to transform it to a unified numerical scale. This solution could eliminate weight bias caused by numerical magnitude, allowing the model training process to focus on the inherent dynamic correlation characteristics of the data.

### 2.3. Flow Information Identification Based on Data Synergy

**Figure 5:**
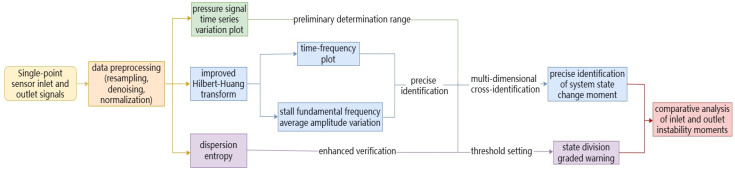
Flow instability graded discrimination design flowchart.

Figure 5 shows the complete process of the flow instability classification and discrimination strategy.

#### 2.3.1. Time Domain Feature Analysis

**Figure 6:**
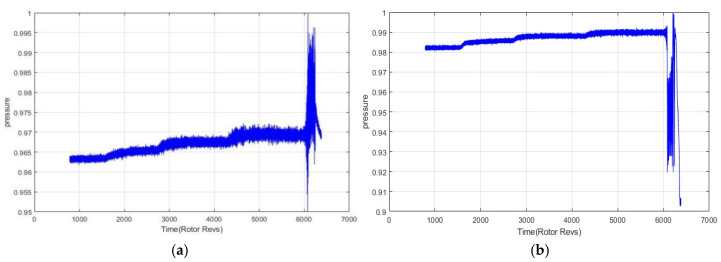
Pressure signals. (**a**) inlet; (**b**) outlet.

After the data preprocessing, the pressure measurements are obtained at inlet and outlet (Figure 6). It can be seen that the compressor is in a stable operating state before the 6000 r. As the operating state approaches the critical interval of rotating stall, the internal flow begins to exhibit large amplitude fluctuations. The stall propagates along the circumferential direction of the compressor. The pressure of the inlet sensor exhibits a significant jump up, while the pressure of the outlet shows a jump-down change. Therefore, it can be preliminarily determined that the approximate moment of instability occurrence is near 6000 r.

#### 2.3.2. Time-Frequency Analysis with Improved Hilbert–Huang Transform

The Hilbert–Huang Transform (HHT) [30], as a high-resolution time-frequency analysis technique, has outstanding applicability in the field of nonlinear signal processing. Its core architecture consists of two synergistic parts: Empirical Mode Decomposition (EMD) and the Hilbert Transform. EMD is an adaptive signal decomposition technique that can decompose complex multi-component signals into several Intrinsic Mode Functions (IMFs) with clear physical meaning, each satisfying the basic condition for defining instantaneous frequency. Then the Hilbert Transform operates on the IMFs obtained from EMD, calculating the instantaneous amplitude and frequency of each mode to characterize the time-frequency features of the signal.

**Figure 7:**
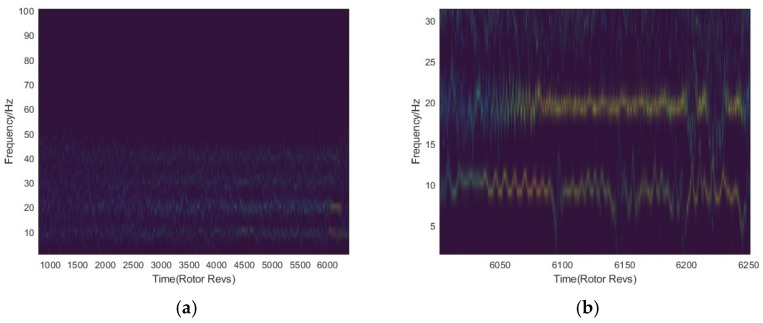
Improved HHT of inlet sensor. (**a**) global; (**b**) local.

**Figure 8:**
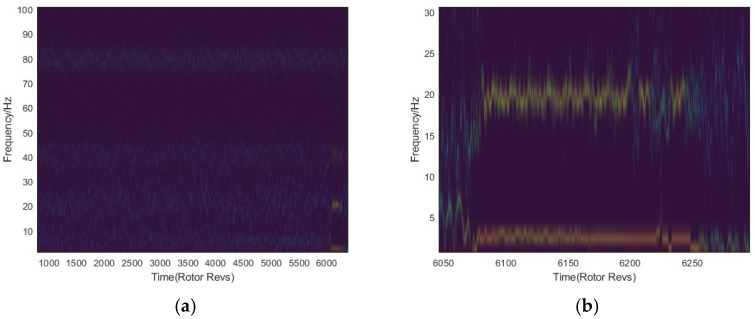
Improved HHT of outlet sensor. (**a**) global; (**b**) local.

Compared with the traditional HHT, the improved Hilbert-Huang Transform replaces EMD with Variational Mode Decomposition (VMD). VMD exhibits a superior robustness when processing complex signals, effectively avoiding the mode mixing problem in traditional decomposition methods. The superiority of VMD over EMD in decomposing unstable and nonlinear signals has been validated by Eriksen [31]. More specifically, Chen provided a direct comparison between VMD and EMD on the fan pressure signals near stall conditions [32]. The results showed that the optimized VMD could well suppress the mode mixing phenomenon existing in EMD, which could greatly enhance the capability to distinguish characteristic frequencies. Based on the preliminary verification [33], the improved Hilbert–Huang Transform not only enhances signal decomposition accuracy but also adapts to the signal characteristics of high-speed compressor. These independent validations collectively support the rationale for the improved Hilbert-Huang transform framework adopted in this study. In summary, as to achieve precise identification of the system instability critical moment, this paper selects the improved Hilbert-Huang Transform method based on VMD. The results with improved HHT are revealed in Figure 7 and Figure 8 for the inlet and outlet sensors.

**Figure 9:**
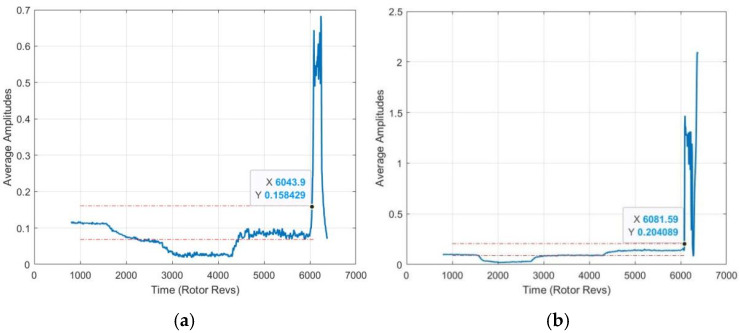
Instantaneous frequency amplitude. (**a**) inlet; (**b**) outlet.

From Figure 7 and Figure 8, it can be found that both inlet and outlet signals show a concentration of a low frequency band around 6050 r with the energy rapidly increasing. Thus, it is preliminarily determined that the compressor experienced stall phenomena around 6050 r. To precisely identify the instability moment, the frequency characteristics of average amplitude are calculated for determination from the time-frequency plots. It is observed that after 6000 r, the inlet signal amplitude shows a significant abnormal fluctuation (Figure 9), intuitively reflecting that the system has entered an unstable state at this time. Based on precise position analysis using time-frequency characteristics and average amplitude change, it is finally determined that the system instability critical moment is at the 6043 r position. Similarly, the signal of the outlet sensor is determined to enter the stall stage at 6081 r.

#### 2.3.3. Stall Classification Strategy by Dispersion Entropy

Dispersion Entropy (DE), as an emerging entropy analysis technique, can quantitatively characterize the complexity and irregularity of the system during the compressor stall evolution. Its core advantage lies in addressing the deficiency of permutation entropy in insufficient utilization of signal amplitude information. Different from traditional methods, DE does not require sorting of embedding dimensions or calculating distances of delay vectors. It also enhances sensitivity to subtle amplitude fluctuations in signals, outperforming sample entropy and permutation entropy in noise suppression, possessing a higher analytical robustness.

The dispersion entropy of a signal x is calculated as follows:

(1) $$DE\left(x,m,c,d\right)=-\sum_{\pi =1}^{c^{m}}P\left(\pi_{v_{0}v_{1}\ldots v_{m-1}}\right)\ln\left[P\left(\pi_{v_{0}v_{1}\ldots v_{m-1}}\right)\right]$$

where m is the embedding dimension, d is the delay time, c is the class function, $P\left(\pi_{v_{0}v_{1}\ldots v_{m-1}}\right)$ is the probability of the dispersion pattern.

**Figure 10:**
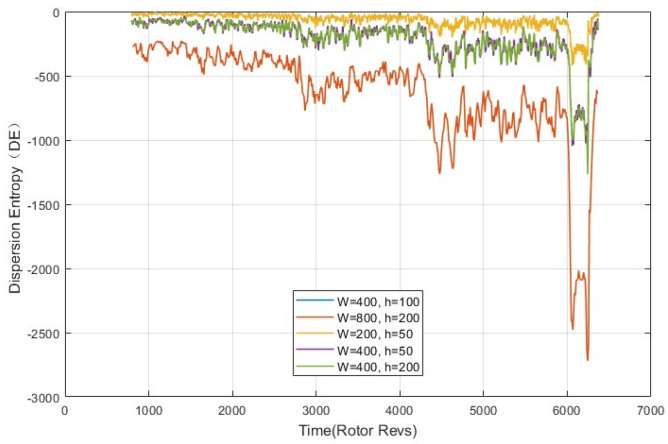
Dispersion entropy variation under different windows and step sizes.

**Figure 11:**
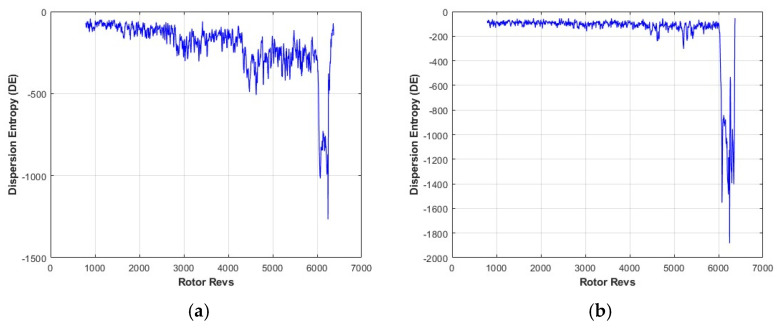
Dispersion entropy variations. (**a**) inlet; (**b**) outlet.

In view of the nonlinear characteristics such as frequency jumps and instantaneous amplitude mutations in rotating stall signals, in order to accurately capture the local dynamic features of the system signals, this paper uses a sliding window mechanism to calculate the dispersion entropy. To determine the window width and step size parameters, multiple parameter combinations of sliding window and step size (*W*, *h*) are set based on the resampling frequency of 4000 Hz, where *W* represents the window width and *h* represents the sliding step size. By observing Figure 10, it is found that when the parameter combination is set to (*W* = 400, *h* = 100), the fluctuation amplitude during the stable stage is small while the entropy response amplitude during the stall stage is significant. Moreover, it could retain more local details while maintaining appropriate computational efficiency. Under this parameter combination, the dispersion entropy results calculated for each window are shown in Figure 11. From the entropy variation curves, it can be seen that as the system state gradually enters the rotating stall, the dispersion entropy index shows a significant downward trend.

This variation trend is physically consistent with the flow structure evolution of rotating stall. Under stable operating conditions, the dynamic pressure signal is dominated by random turbulent pulsations in the flow passage, featuring high irregularity and thus a high dispersion entropy value. As the compressor approaches the stall boundary, periodic stall cells and modal waves gradually form in the flow field, which enhances the regularity of the pressure signal and reduces its complexity, leading to a continuous decrease in dispersion entropy.

From the figures above, it can be observed that when the compressor is in stable operation, the dispersion entropy index remains within a relatively stable range. And the fluctuation amplitudes of the entropy values calculated by each sliding window are extremely small. As the system operating state gradually approaches the stall boundary, the characteristics such as frequencies contained in the flow field become significantly more complex, the dispersion entropy exhibits a significant downward trend. When the system formally enters the rotating stall, the decline amplitude of this entropy value further expands. Based on this variation pattern, multi-level progressive warning thresholds are set:

(2) $$\varepsilon_{1}=\mu_{stable}-3\sigma_{stable}$$

(3) $$\varepsilon_{2}=\mu_{global}-3\sigma_{global}$$

In this strategy, $\varepsilon_{1}$ represents the first-level warning threshold. When the dispersion entropy falls below this threshold for the first time, the system enters the early stall precursor stage. $\varepsilon_{2}$ represents the second-level warning threshold. When the entropy value continues to decline and falls below this threshold, it means that the system has formally entered the rotating stall stage. $\mu_{stable}$ denotes the mean dispersion entropy during the stable stage, and $\mu_{global}$ denotes the global mean dispersion entropy. $\sigma_{stable}$ denotes the standard deviation of the dispersion entropy during the stable stage, and $\sigma_{global}$ denotes the global standard deviation of the dispersion entropy.

**Figure 12:**
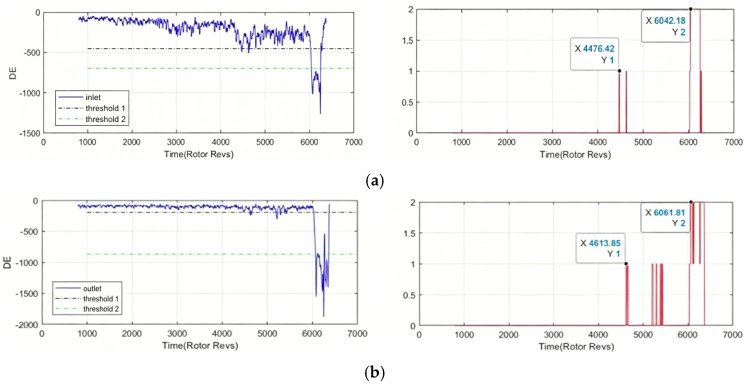
Dispersion entropy warning result. (**a**) inlet; (**b**) outlet.

Combined with the dispersion entropy curves from the inlet and outlet measurement points, the division of the compressor instability process can be clearly defined by setting warning flags of “0 for stable state, 1 for below the first-level warning threshold, and 2 for below the second-level warning threshold”. In Figure 12, the inlet signal first falls below the first-level threshold at 4476 r, and the flag switches to 1, indicating that the system begins to experience small disturbances and enters the stall precursor stage. Around 6042 r, the entropy value decreases significantly, triggering the second-level threshold. The flag switches to 2, indicating that the system formally enters the stall stage at this moment, with a rapid transition from precursor stall to rotating stall. The outlet signal exhibits small disturbances around 4613 r. Compared with the stable stage, the entropy fluctuation range expands, triggering the first-level warning for the first time. And it enters the stall stage at 6061 r. The precise positions of identification at each stage are revealed in Table 2.

Based on the variations of pressure signals, the improved HHT, and the dispersion entropy, a multi-dimensional feature cross-validation of the system is performed to achieve precise localization of the system instability. According to the defined discrimination thresholds, three stages are successfully divided as stable stage, stall precursor stage, and formal stall stage.

## 3. Deep Learning Modeling Embedded with Mode Decomposition Optimization

### 3.1. Dung Beetle Optimization

The Dung Beetle Optimizer (DBO) [34] is a new type of swarm intelligence optimization algorithm whose bionic inspiration mainly comes from the behaviors of dung beetle populations in nature, specifically including rolling balls, dancing, foraging, stealing, and reproduction. Its core advantage lies in achieving a dynamic balance between global exploration and local exploitation during the optimization process. This characteristic gives DBO significant advantages in both convergence speed and optimization accuracy, avoiding the common drawbacks of falling into local optima and slow convergence in traditional algorithms.

The core function of VMD is to decompose the input signal, deconstructing the original signal *f* into several IMFs with different center frequencies. The number of modes *K* and the mode frequency bandwidth control parameter (quadratic penalty term) α are the key parameters for VMD decomposition. To avoid the limitations of manual setting, this paper uses DBO to find the optimal parameter combination matching the system characteristics.

In the signal processing of VMD, envelope entropy, as a statistical quantification index based on the envelope signal, is used to characterize the complexity and uncertainty of the signal. By deconstructing the envelope characteristics of the original signal, it can effectively capture the characteristic information contained in the signal at different scale levels. Compared with other entropy measurements, envelope entropy is demonstrated to have significant advantages in scale adaptability, noise suppression, and computation efficiency. This enables it to efficiently process multi-scale, complex signals, and better match the differentiated features of each mode function within the VMD decomposition framework.

Based on this, this paper selects the minimum envelope entropy as the fitness function for the DBO algorithm to optimize VMD parameters. When the decomposed IMFs have a high proportion of noise components, the envelope entropy is at a high level. Conversely, when the IMFs components are enriched with effective features and adequate noise suppression, the envelope entropy value decreases significantly.

Let a signal x(t) be decomposed by VMD into multiple intrinsic mode functions $u_{k}(t)(t=1,2,\ldots \;,k)$, and the envelope of each mode function be $A_{k}(t)$. The envelope entropy $E_{p}$ can be defined as follows:

(4) $$E_{p}=-\sum_{k=1}^{K}P_{k}\log P_{k},P_{k}=\frac{\int_{t_{1}}^{t_{2}}A_{k}\left(t\right)dt}{\sum_{j=1}^{K}\int_{t_{1}}^{t_{2}}A_{j}\left(t\right)dt}$$

where $P_{k}$ is the probability distribution of the envelope of the k-th mode function, and $A_{k}$ is the envelope of the k-th mode function $\mu_{k}(t)$.

During the parameter optimizations, the DBO parameters are set in Table 3.

**Figure 13:**
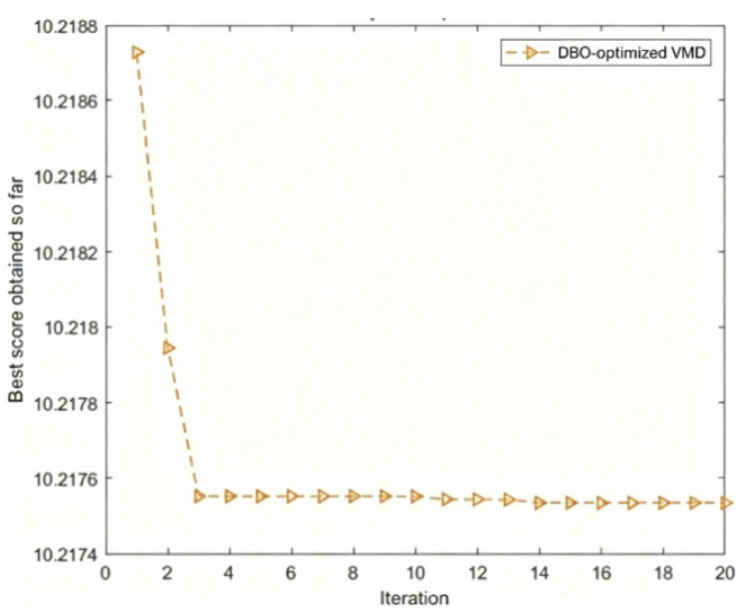
Optimization iteration of minimum envelope entropy.

The iterative optimization process of the DBO algorithm is shown in Figure 13 below. Throughout the entire parameter optimization, the minimum envelope entropy fitness function value exhibits a continuous decline and stabilizes toward convergence after the third iteration, essentially reaching a stable state. At this point, the minimum envelope entropy is 10.2175, and the optimal parameter combination of the penalty factor and the number of IMFs $\left(\alpha ,K\right)$ is (2430, 10).

### 3.2. Long Short-Term Memory Network

**Figure 14:**
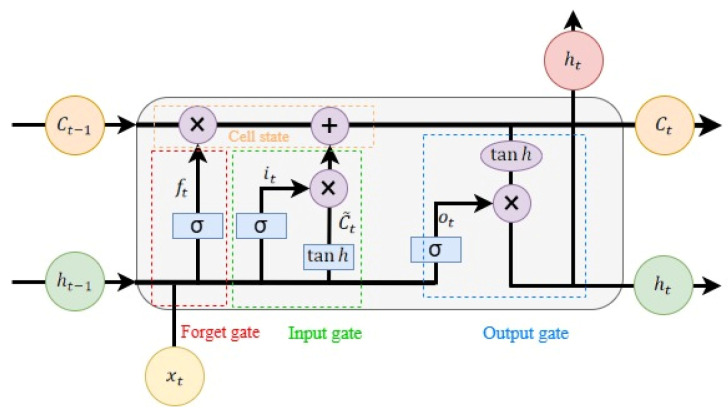
Structure of LSTM.

To avoid feature submersion caused by mixed training of multiple components, a layered modeling strategy is adopted. An independent LSTM sub-network (Figure 14) is trained for each IMF component $\mu_{k}(t)$, and finally, the prediction results of each component are superimposed in the feature fusion layer.

LSTM controls information flow through memory cells and gating mechanisms, with the calculation process as follows.

Forget gate:

(5) $$f_{t}=\sigma \left(W_{f}\cdot \left[h_{t-1},x_{t}\right]+b_{f}\right)$$

It determines which information from the previous time step needs to be retained and which needs to be forgotten.

Input gate:

(6) $$i_{t}=\sigma \left(W_{i}\cdot \left[h_{t-1},x_{t}\right]+b_{i}\right)$$

It is responsible for selecting the new information input at the current moment.

Candidate cell state:

(7) $${\tilde{C}}_{t}=\tan h\left(W_{c}\cdot \left[h_{t-1},x_{t}\right]+b_{c}\right)$$

Cell state update:

(8) $$C_{t}=f_{t}\cdot C_{t-1}+i_{t}\cdot {\tilde{C}}_{t}$$

Output gate:

(9) $$o_{t}=\sigma \left(W_{o}\cdot \left[h_{t-1},x_{t}\right]+b_{o}\right)$$

It determines which information in the memory cell at the current moment needs to be output as the hidden state, and influences the output of the current time step.

Hidden state:

(10) $$h_{t}=o_{t}\cdot \tan h\left(C_{t}\right)$$

### 3.3. VMD_DBO_LSTM Model Construction

**Figure 15:**
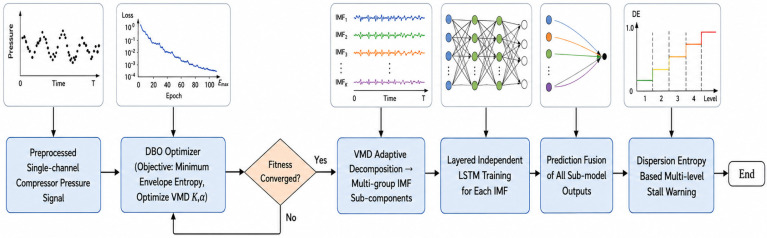
VMD_DBO_LSTM model flowchart.

To improve the accuracy and reliability of rotating stall prediction, a VMD_DBO_LSTM coupled prediction model is proposed, fully combining the multi-scale feature separation advantages of VMD and the temporal dependency-capturing capability of the LSTM. This model takes “Signal Decomposition-Layered Components Prediction-Feature Fusion” as its core logic to precisely capture the nonlinear and highly fluctuating characteristics of sensor-collected data. It adopts DBO to optimize the two key parameters of VMD, solving the deficiency of traditional models in adapting to the complex dynamic stall signals and the adverse effects caused by human intervention. Simultaneously, it utilizes a layered LSTM architecture to target the learning of the patterns of each decomposed IMF, effectively avoiding the problem of insufficiently capturing complex signal features. The flowchart of this model is shown in Figure 15.

To verify the superiority of the proposed model, this paper designs multiple sets of comparative experiments based on inlet measurement. The specific settings are as follows. The input sequence length is set to 200 steps, the hidden layer dimension is 128, the batch size is fixed at 128, the optimizer is Adam, and the learning rate is set to 0.001.

The comparison models include the LSTM model, VMD_LSTM model, and VMD_DBO_LSTM model. The experiments evaluate the performance of each model from the perspective of accuracy and prediction capability, focusing on the compressor stall detection effect. This study uses four performance indicators to comprehensively evaluate the models: Mean Absolute Error (MAE), Root Mean Square Error (RMSE), Mean Absolute Percentage Error (MAPE), and the coefficient of determination (R^2^).

Through the unified indicator comparison, it is found that the layered LSTM model after introducing VMD decomposition has a higher feature capturing capability, proving the key role of VMD in decomposing complex stall signals, stripping noise, and highlighting precursor features. On this basis, by introducing the DBO algorithm to precisely optimize VMD parameters, the prediction accuracy can be further improved by achieving targeted learning of each IMF component.

The experiment results (Table 4) show that through the collaborative design of decomposition and layered learning, the VMD_DBO_LSTM model reduces MAE by 64% ($\mathrm{reduction}\;\mathrm{rate}=\frac{{\mathrm{MAE}}_{\mathrm{LSTM}}-{\mathrm{MAE}}_{\mathrm{VMD}\_\mathrm{DBO}\_\mathrm{LSTM}}}{{\mathrm{MAE}}_{\mathrm{LSTM}}}\times 100\%$) and improves R^2^ by 4.46% ($\mathrm{improvement}\;\mathrm{rate}=\frac{R_{\mathrm{VMD}\_\mathrm{DBO}\_\mathrm{LSTM}}^{2}-R_{\mathrm{LSTM}}^{2}}{R_{\mathrm{LSTM}}^{2}}\times 100\%$) compared with the baseline LSTM model, outperforming other models in all four indicators. It can accurately capture the precursor features and evolution of rotating stall, improving the prediction accuracy and stability of complex stall signals.

**Figure 16:**
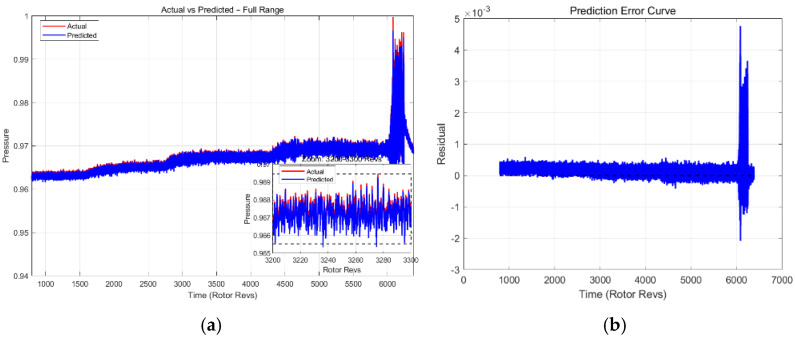
Model performance. (**a**) prediction results; (**b**) errors.

**Figure 17:**
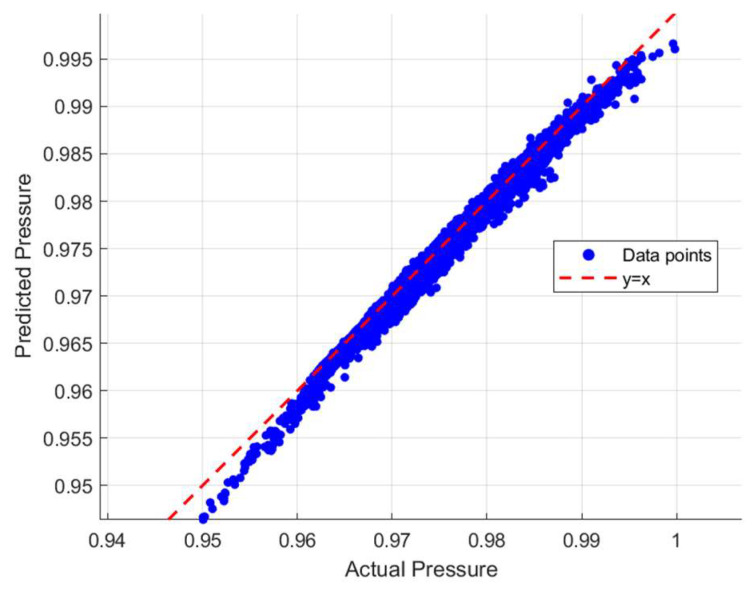
Model scatter fitting plot.

Based on the established compressor stability model, this paper carries out unsteady flow prediction and compares the results with experiment data. From the time series comparison plot in Figure 16, it is intuitively found that the predicted values remain highly consistent with the original data. The error is mostly stable near zero, with only fluctuations occurring during the flow instability phase. The maximum absolute error does not exceed 0.005. The Actual-Predicted scatter fitting plot of Figure 17 shows that the linear correlation between the model predicted values and the actual values is extremely in consistency. Therefore, the VMD_DBO_LSTM layered time series fusion model can accurately capture the pressure variation characteristics in the compressor unsteady flow field.

### 3.4. Stall Prediction by Dispersion Entropy with Multi-Level Discrimination Strategy

**Figure 18:**
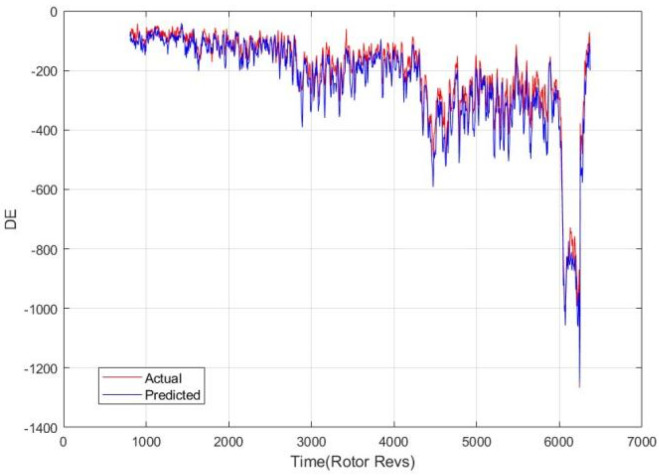
Comparison of dispersion entropy.

For the VMD_DBO_LSTM prediction results, the dispersion entropy performs dynamic characteristic analysis. To ensure comparability between the prediction results and the actual results, the sliding window length is still set to 400 data points, and the sliding step is set to 100 steps. The core parameter configuration of the dispersion entropy is set as embedding dimension, number of classes, and time delay. The results in Figure 18 indicate that the dispersion entropy curves of the predicted values and the true values exhibit a highly consistent evolutionary trend, and the mutation time nodes basically coincide completely. When a stall occurs, the dispersion entropy drops significantly. After the occurrence of rotating stall, its value is significantly lower than the level under normal operating conditions. This confirms the sensitive capturing ability of dispersion entropy for the nonlinear characteristics of rotating stall.

**Figure 19:**
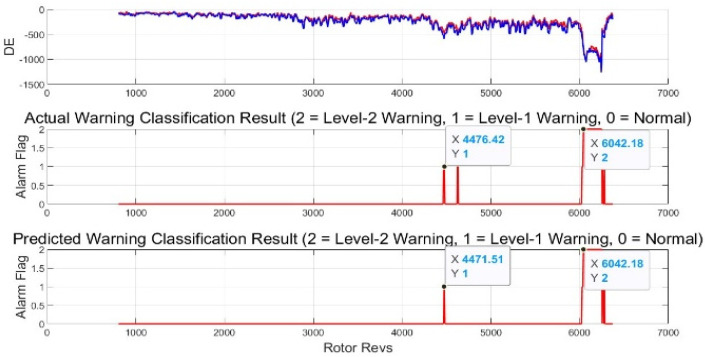
Comparisons between predicted and true values.

To achieve precise identification of stall precursors, the procedure is performed based on the progressive warning threshold proposed earlier. From Figure 19, it can be seen that the corresponding predicted stall precursor is at the 4471 r, allowing the detection of abnormal fluctuations in the system at 1572 r (corresponding to 8 s) in advance. The moment of entering the full stall stage is 6042 r. According to the threshold discrimination, the actual and predicted warning moments are the same and are highly consistent with the 6043 r identified from the earlier measured data. It is fully verified that the reliability of the threshold setting in this study for determining the compressor instability critical point.

## 4. Multi-Source Information Cross-Fusion Deep Learning Model

### 4.1. Cross-Attention Mechanism

The core function of the cross-attention mechanism is to build dynamic associations between different modal data or multiple differentiated features within the same modality, thereby completing the deep and efficient fusion of multi-source features. Unlike traditional self-attention mechanisms, which mostly focus on temporal and spatial dependencies within a single modality, the cross-attention mechanism supports information interaction between two or more input sequences. It enables the reinforcement of information exchange and feature complementarity between different features and modalities.

The operation process of the cross-attention mechanism is based on three core matrices: Query (Q), Key (K), and Value (V). Through a set of linear mapping layers, one group of features is mapped to the query vector Q. Then, through another independent set of linear mapping layers, another group of features is projected into the key vector K and the value vector V, respectively. The goal of this projection operation is to map the original features into different feature subspaces, enhancing the understanding ability of information. The subsequent process is to calculate the similarity between the query vector Q and the key vector K to obtain the weight distribution of each position in the sequence. This weight can intuitively reflect the degree of correlation dependence between the two groups of features. Then, the SoftMax function is used to normalize the similarity results, obtaining a standardized weight map. Finally, the weight map is weighted with the value vector V, outputting the fused feature map, thus completing the entire process of feature fusion. The formula is set as follows:

(11) $$Attention\left(Q,K,V\right)=softmax\left(\frac{QK^{T}}{\sqrt{d_{k}}}\right)V$$

where Q typically originates from the target modality, and K and V represent feature vectors from other modalities, $d_{k}$ is the scaling factor.

### 4.2. Multi-Scale Feature Extraction Based on CA_BiLSTM

When stall occurs inside a compressor, the flow field will exhibit circumferential or axial flow instability phenomena. Different locations within the compressor will produce differentiated characteristics. If only a single sensor pressure data is relied upon, it is highly prone to problems such as misjudgment and missed detection of early stall signals. Therefore, based on the previous research, an instability detection model based on the pressure feature fusion of inlet and outlet dual channels is proposed.

Targeting the temporal correlation and physical constraint characteristics of inlet and outlet aerodynamic data, a time series data fusion model integrating the cross-attention mechanism and bidirectional LSTM is constructed. This model takes “Time Series Feature Extraction-Dynamic Weight Allocation-Physical Constraint Optimization” as its core logic, autonomously learning the spatiotemporal features of dual-channel pressure data through deep learning. It abandons the subjectivity and limitation of manual selection, achieving deep fusion of multi-source information, and generating fused features capable of comprehensively characterizing the stall state.

This fusion model consists of a data preprocessing module and a five-layer network structure. Each layer progressively advances around three main objectives: time series feature extraction, dynamic weight generation, and fusion result optimization. The specific structure is as follows.

1. Feature Extraction. A shared Conv1D layer and ReLU activation function are adopted to synchronously extract the local time series features of the inlet and outlet data through weight sharing. With capturing fine-grained information such as high-frequency pressure pulsations, L2 regularization is introduced to suppress overfitting.
2. Feature Optimization. A batch normalization layer accelerates convergence and alleviates gradient vanishing, combined with a Dropout layer that randomly masks some feature nodes to improve the generalization capability.
3. Time Series Modeling. A shared bidirectional LSTM layer is employed to simultaneously capture the forward and backward temporal dependencies of the inlet and outlet data, deeply mining the slowly varying characteristics of stall precursors and expanding unidirectional time series into bidirectional fusion features.
4. Weight Generation. Through the cross-attention module, bidirectional interaction and dynamic weight adaptive allocation of the inlet and outlet features are achieved. After smoothing by a 3rd-order mean convolution, a Lambda layer is used to hard-code the physical constraint that the sum of weights equals 1.
5. Fusion Optimization. Weighted fusion generates an initial fused value, which is then refined and corrected by a residual optimization block (two layers of Conv1D, batch normalization, residual connection). Finally, dual-branch results of the fused feature and the dynamic weights for the inlet and outlet are output.

**Figure 20:**
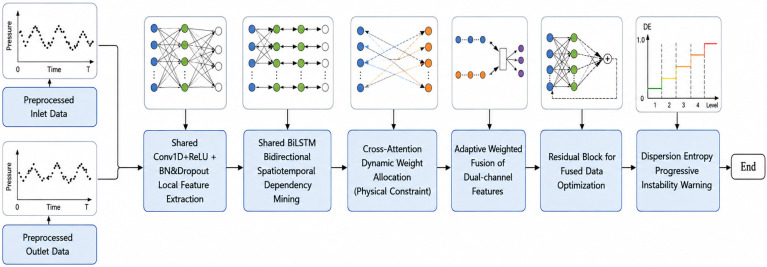
CA_BiLSTM model flowchart.

The specific path of this model is indicated in Figure 20.

To simultaneously optimize the model accuracy and the rationality of the weight allocation, this paper adopts a multi-task joint loss function and a combined training strategy to optimize the CA_BiLSTM model. This optimization strategy uses the MSE of the fusion result as the core loss, and the MSE of the weight fitting as the auxiliary loss. And it is paired with the Adam optimizer, cosine annealing learning rate scheduling, and an early stopping strategy for collaborative training. Among them, the Adam optimizer sets the initial learning rate to 0.0008 and incorporates gradient clipping to ensure the stability of gradient update. The cosine annealing scheduling strategy adopts a dynamic adjustment mode of 5-epoch warm-up followed by 45-epoch cosine decay. It ensures a faster convergence speed in the early stage of model training and achieves a refined weight optimization in the later stage, thus balancing the convergence efficiency and prediction accuracy. The early stopping strategy employs the validation set fusion loss as the monitoring indicator, with patience set to 15. When the validation set loss does not decrease significantly for 15 consecutive epochs, training is immediately terminated. And the model weights with the best generalization ability are retained, effectively avoiding the problem of model overfitting.

During the prediction phase of the model, for the time series samples generated by the sliding window, a reconstruction algorithm is performed mean fusion processing on the prediction results of overlapping windows to eliminate the prediction bias caused by window overlap. Finally, a full-length fused feature sequence and dynamic weight sequence are output, which are equal in length to the original data. It ensures the temporal integrity of the fusion result while quantifying the credibility of the inlet and outlet data through dynamic weights. It provides an intuitive and reliable quantitative basis for real-time monitoring and fault diagnosis of the compressor operating status.

To verify the performance of the CA_BiLSTM model constructed in this paper, the analysis is conducted from three aspects: weight characteristic, fusion effect, and quantitative indicators based on the full-cycle operation.

**Figure 21:**
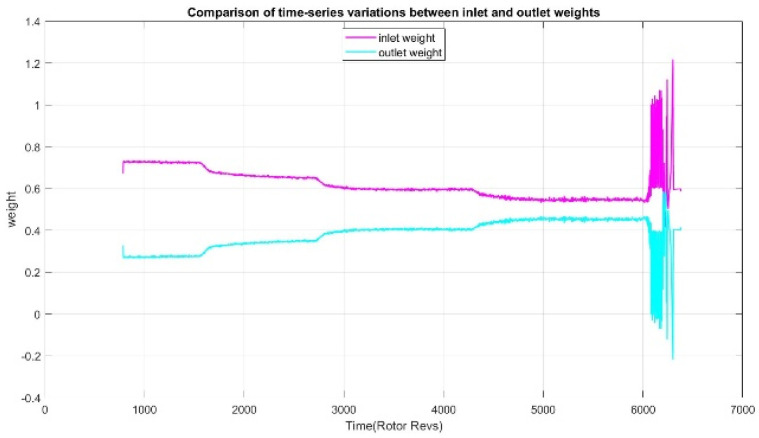
Weight variation curve.

**Figure 22:**
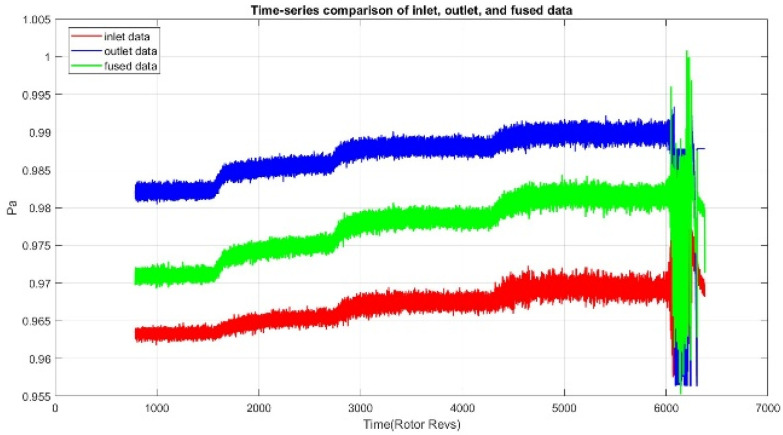
Comparisons of fusion data.

1. Verification of weight allocation characteristic. The weight variation curve (Figure 21) shows that during the stable stage, the inlet weight stabilizes in the range of 0.5~0.8, while the outlet weight gradually rises from 0.3 to 0.5. The sum of weights is basically maintained around 1, verifying the effectiveness of the weight normalization physical constraint. Through the previous research on the inlet and outlet data, it is found that compared with the outlet data, the inlet information is simpler and clearer, while the outlet contains more complex and chaotic information. Therefore, the inlet weight being overall higher than the outlet weight conforms to prior expectations. The extreme switch of the inlet weight surging and the outlet weight plummeting during the mutation stage is a typical response of the model to the compressor stall precursor. It is highly consistent with the engineering laws of aerodynamic instability. Meanwhile, the drastic fluctuation of weights during the mutation stage can serve as a warning feature for compressor stall, providing an intuitive quantitative basis for subsequent fault diagnosis. It is also proved the dynamic response capability and engineering practicality of the cross-attention mechanism in time-series data fusion.

2. Time series feature analysis of fused data. It can be seen from Figure 22 that the variation pattern of the fused data is highly consistent overall with the original inlet and outlet data, verifying the feature retention capability of this model. During stable operation (0~6000 r), the fused data is always located between the original inlet and outlet data, and the fluctuation amplitude is significantly smaller than the two original curves. This indicates that the model achieves a balance between noise suppression and feature retention through dynamic weight allocation, effectively improving the robustness of the data. The change trend of the fusion value completely corresponds to the weight allocation pattern, verifying the rationality of the dynamic weight design. During the mutation stage (6000 r~6500 r), the all original data exhibit violent fluctuations, while the fluctuation amplitude of the fusion data is significantly compressed, reflecting the anti-interference capability in an extreme scenario. The complementary mechanism of dynamic weights avoids excessive interference from the single-channel extreme values on the fusion result, ensuring the stability of the output.

3. Model performance quantification. The final model training loss is as low as 0.000202, indicating the deviation between the fusion result and the true value is extremely small, further verifying the model fitting accuracy and reliability. From the perspective of signal quality analysis, the Signal-to-Noise Ratio (SNR) of the original inlet and outlet experiment data is maintained at approximately 39.3 dB. After dual-source fusion, the SNR of the fused data increases to 48.2 dB. This result indicates that the dual-source fusion strategy effectively achieves the superposition of effective signals and the suppression of single-channel noise, significantly improving signal quality. From the perspective of feature integrity, while achieving noise suppression, the Feature Retention Rate (FRR) of the fused data reaches 84.02%. It is effectively ensured that key features, such as stall precursors, are not excessively smoothed or lost. In addition, the sequence length of the fused data is completely consistent with the original data. And there is no phase shift, ensuring the integrity of the time series structure, making it applicable to the monitoring and analysis of the compressor full-cycle operating status.

In summary, the CA_BiLSTM dual-channel model does not take merely reducing prediction error as its core objective but achieves a fundamental improvement in model robustness, anti-interference performance, and rotating stall feature identification accuracy. The single-sensor models that only rely on information from a single measurement are susceptible to local noise interference and signal distortion, leading to problems such as misjudgment of the stall state and missed detection of early precursor signals. The fusion model proposed in this chapter realizes dynamic weight allocation of inlet and outlet data through cross-attention mechanism. It can adaptively enhance the effective feature information of high-credibility channels while suppressing noise interference from abnormal channels. This model can achieve effective smoothing of noise signals under stable compressor operating conditions and maintain high feature response sensitivity during the stall mutation stage. Through information complementarity and redundancy enhancement, it effectively overcomes the limitation of single-sensor monitoring information unintegrity.

### 4.3. Stall Detection with Dispersion Entropy

**Figure 23:**
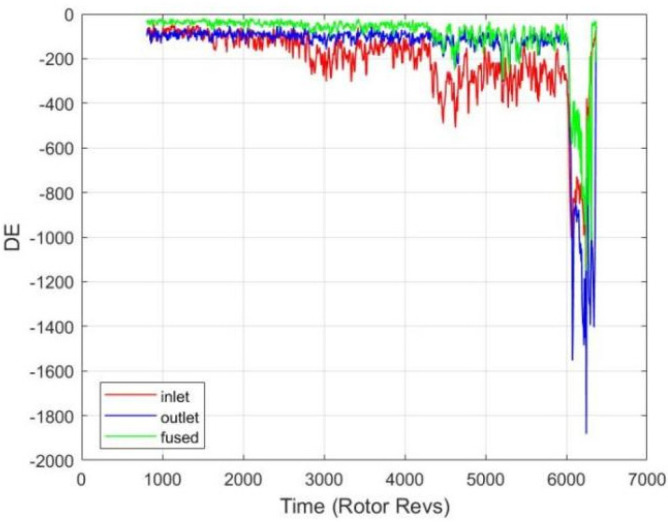
Dispersion entropy comparisons of fusion data.

According to the results of the multi-source fusion information driven by the CA_BiLSTM model, the dispersion entropy algorithm is applied to detect the state of compressor. And the discrimination results are compared horizontally with the results based on the earlier identification data. To ensure the comparability of results from different data sources, the same parameter configuration is used as in the previous study. The dispersion entropy curves are revealed in Figure 23.

During the stable stage, the entropy values of the three types differ, but the time location of the sudden drop in entropy when stall occurs is highly consistent. It is indicated that both single-sensor and fusion data can effectively capture stall events. The entropy fluctuation amplitude of the fusion data lies between those of the inlet and outlet, confirming that multi-source fusion can suppress the interference of local random noise. It has improved the stability of state monitoring, thereby enhancing sensitivity and reliability of early warning.

**Figure 24:**
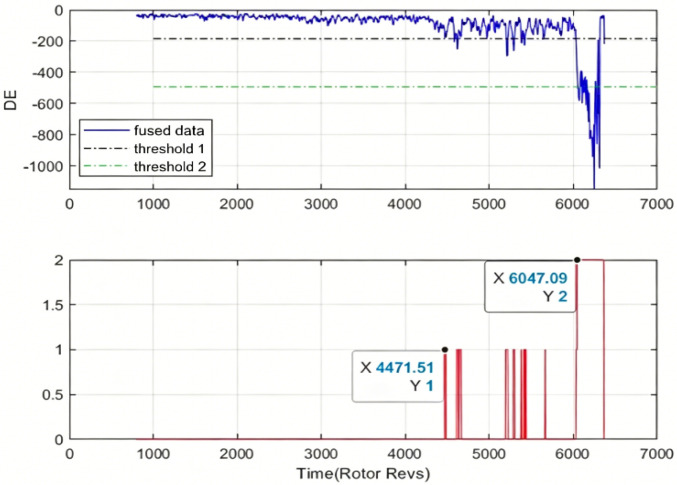
Multi-level discrimination results of fusion model.

The dispersion entropy threshold progressive discrimination method is employed to classify the status of the fusion data, as shown in Figure 24. It is detected a slight abnormal fluctuation in the system at the 4471 r (1580 r ahead of stall occurrence, corresponding to 8.05 s), marking the system transition from the stable stage to the stall precursor stage. Further discrimination indicates that the moment is the 6047 r when the system formally enters the stall stage.

Based on the integrated comparisons of all the above results in Table 5, it is found that the two warning locations of the CA_BiLSTM model are highly close to those of inlet experiment data and VMD_DBO_LSTM model, with no significant deviation. In this experiment, the compressor rotating stall occurs at the rotor tip of the first stage, which confirms that the detection performance of the inlet measurement is better than that of the outlet measurement. However, the origin location of stall cell in a multistage compressor is affected by operating conditions. It can be distributed in different regions for different compressor designs. This also leads to inherent limitations in the detection scheme using a single sensor measurement. If the measure arrangement does not match the actual stall location, it may cause failure in capturing stall precursors, leading to problems such as delayed warning, reduced identification accuracy, or even missed and false judgments. In the subsequent research, it can target the operating condition dependency of the stall location by deploying multi sensors. Combined with multi-source data fusion algorithms, it is to build an adaptive measurement selection and weight allocation model, thereby achieving precise detection and early warning under different stall locations, fundamentally solving the detection limitations of a single sensor.

## 5. Discussion

It should be noted that the current verification of the proposed framework is conducted under a constant speed condition, which covers the main application scenarios of compressor stability monitoring. Nevertheless, from a mechanistic perspective, the proposed framework exhibits promising adaptability to off-design operating conditions and different sensor configurations. The dispersion entropy-based thresholding strategy is inherently scale-invariant and does not depend on absolute operating parameters, as it characterizes the relative complexity variation of the pressure signal during stall evolution.

Meanwhile, the VMD_DBO_LSTM prediction model, with its adaptive parameter optimization via the DBO algorithm, can be re-optimized for signals under new operating conditions without manual empirical tuning, thereby accommodating varying frequency distributions under different rotational speeds. The CA_BiLSTM fusion model, relying on dynamic cross-attention weight allocation rather than fixed coefficients, can also adapt to changes in the relative credibility of inlet and outlet signals under transient conditions. And it is compatible with extended multi-sensor array layouts after structural adjustment. These intrinsic adaptive mechanisms suggest that the proposed framework is likely to maintain robust detection performance across a range of operating conditions and measurement schemes.

The proposed method is primarily intended for early warning of rotating stall under near-stall conditions, which represents the most critical scenario for compressor stability protection. However, it must be acknowledged that the current experimental validation does not cover variable-speed ramping, load transients, inlet distortion conditions, or diverse sensor arrangements. Systematic validation under these extended regimes—including quantitative assessment of warning advance time, false alarm rate, and model generalization error—remains a critical direction for future work. Specifically, the optimization of the dispersion entropy threshold under varying rotational speeds and the fine-tuning of the DBO parameter search space for transient spectral features will be the primary focuses in subsequent research, as well as the incorporation of adaptive sliding window strategies for CA_BiLSTM under rapidly changing flow conditions to further expand the engineering application of the framework.

## 6. Conclusions

As the core support for active control systems, the precise detection and early warning of stall signals are the kernel tasks in the field of compressor instability identification. Targeting the nonlinearity of signals in a complex flow field, a series of technical methods and deep learning model architectures are proposed by combining multi-dimensional feature analysis in the time domain, time-frequency domain, entropy, attention mechanism, and intelligent optimization algorithm. It is formed as an integrated “Identification-Fusion-Prediction” stall warning solution under the premise of ensuring the reliability and robustness of instability identification. The main conclusions are as follows:

1. Multi-dimensional feature cross-identification and progressive graded early warning strategy. Aiming at the problem of inaccurate single-feature identification, a cross-identification scheme is proposed by integrating time domain, time-frequency domain, and dynamic entropy features. Under the construction of a multi-dimensional feature verification system, it is determined the precise instability locations for inlet and outlet signals are 6043 r and 6081 r, respectively. Based on the dispersion entropy algorithm, the stall process is divided into three stages, achieving a progressive graded early warning. The maximum advance warning inlet and outlet signals reach 7.98 s and 7.47 s, respectively.
2. VMD_DBO_LSTM deep learning modeling for rotating stall. To break through the bottleneck of traditional fusion based on chaotic data feature correlation, a coupled prediction model combined with VMD and LSTM is proposed. By introducing DBO with minimum envelope entropy as the optimization objective, adaptive optimization of the key VMD parameters is achieved. This solves the problems of difficult optimization of model parameters and insufficient mining of temporal features, significantly improving the accuracy of prediction. Experiments show that the model coefficient of determination reaches over 0.99. Combined with the dispersion entropy and three-sigma criterion, the maximum warning advance can be reached as 1571 r.
3. Multi-source information fusion deep learning model with CA_BiLSTM. Targeting the spatiotemporal differences of multi-source from inlet and outlet sensors, the CA_BiLSTM fusion model is proposed with integrating dynamic weight adaptive allocation, physical constraint hard-coding, and residual optimization techniques. To overcome the limitation of static fusion, a weight sharing mechanism and a cross attention dynamic weight allocation mechanism are used to ensure the fusion process is in line with the aerodynamic mechanism. Experiments show that the final model training loss is 2.02 × 10^−4^. Combined with the dispersion entropy threshold, stall precursor can be detected as early as 8.05 s in advance.

## Figures and Tables

**Table 1:** Experiment rig specifications.

Indicator	Value
Maximum power (MW)	5
Maximum speed (rpm)	15,000
Maximum main flow rate (kg/s)	60
Inlet temperature	Room temperature
Inlet pressure (MPa)	0.06~0.1
Maximum exhaust temperature (°C)	300
Maximum exhaust pressure (MPa)	0.4

**Table 2:** Multi-level discrimination warning.

Detection Sensor	Inlet	Outlet
Stall location	6043 r	6081 r
1st warning location	4476 r	4613 r
2nd warning location	6042 r	6061 r
1st warning advance time	7.98 s	7.74 s
2nd warning advance time	0.005 s	0.102 s

**Table 3:** DBO parameter settings.

Parameter	Value
Population size	25
Maximum iterations	20
Number of variables	2
Penalty factor α range	[500, 2500]
Number of IMFs K range	[3, 10]

**Table 4:** Comparisons of model performance.

Model	MAE	RMSE	MAPE	R^2^
LSTM	5 × 10^−4^	1.1 × 10^−3^	6.1 × 10^−6^	0.9503
VMD_LSTM	4 × 10^−4^	8.9 × 10^−4^	4.1 × 10^−6^	0.9679
VMD_DBO_LSTM	1.8 × 10^−4^	2.8 × 10^−4^	1.8 × 10^−6^	0.9927

**Table 5:** Multi-level discrimination warning times.

Detection Sensor	Stall Location	1st Warning Location	2nd Warning Location	1st Warning Advance Time	2nd Warning Advance Time
Inlet	6043 r	4476 r	6042 r	7.98 s	0.005 s
Outlet	6081 r	4613 r	6061 r	7.47 s	0.102 s
VMD_DBO_LSTM	6043 r	4471 r	6042 r	7.98 s	0.005 s
CA_BiLSTM	6051 r	4471 r	6047 r	8.05 s	0.020 s

## Data Availability

The data that support the findings of this study are available from the corresponding author upon reasonable request.

## References

[1] Krejsa T., Němec V., Hrdinová L. (2018). Causes of Aviation Accidents and Incidents Especially with Engine Failure. Transport Means: Proceedings of the International Conference.

[2] Qiao Y., Chu W., Chen J., Sun Z., Wang K., Zhang H. (2025). Discovery, interpretation, and model-based judgment of a hidden operating condition during the process from compressor stall to surge induced by inlet distortion. Aerosp. Sci. Technol..

[3] Fang Y., Sun D., Xu D., He C., Sun X. (2023). Rapid prediction of compressor rotating stall inception using Arnoldi eigenvalue algorithm. Am. Inst. Aeronaut. Astronaut. J..

[4] Zanotti S., Ceschini D., Ferlauto M. (2024). AI-Based Detection of Surge and Rotating Stall in Axial Compressors via Dynamic Model Parameter Estimation. Fluids.

[5] Ji J., Hu J., Ma S., Xu R. (2022). A Computational Method of Rotating Stall and Surge Transients in Axial Compressor. Energies.

[6] Mohammad A., Yahya A., Mohammad N.N. (2022). Estimations of Compressor Stall and Surge Using Passage Stall Behaviors. Machines.

[7] Tu B., Zhang X., Hu J., Zhong M., Xiong B. (2021). Analysis methods for aerodynamic instability detection on a multistage axial compressor. Int. J. Aerosp. Eng..

[8] Qiu X., Liu Z. (2021). Stall warning of axial compressor using spatial FFT and combined analysis of multiple statistical parameters. J. Phys. Conf. Ser..

[9] Shabbir M., Liang X., Chakrabarti S. (2020). An ANOVA-based fault diagnosis approach for variable frequency drive-fed induction motors. IEEE Trans. Energy Convers..

[10] Chen Z., Liu C., Ding S.X., Peng T., Yang C., Gui W., Shardt Y.A.W. (2020). A just-in-time-learning-aided canonical correlation analysis method for multimode process monitoring and fault detection. IEEE Trans. Ind. Electron..

[11] Xu L., Pennacchi P., Chatterton S. (2020). A new method for the estimation of bearing health state and remaining useful life based on the moving average cross-correlation of power spectral density. Mech. Syst. Signal Process..

[12] Chen J., Li Z., Pan J., Chen G., Zi Y., Yuan J., Chen B., He Z. (2016). Wavelet transform based on inner product in fault diagnosis of rotating machinery: A review. Mech. Syst. Signal Process..

[13] Fan Z., Du J., Liu Y., Ba D., Xu X., Zhang M., Zhu Z. (2026). Instability warning method via fast wavelet transform in a high-speed axial flow compressor: From surge precursors to early warning. Aerosp. Sci. Technol..

[14] Lou F., Key N. (2020). Compressor Stall Warning Using Nonlinear Feature Extraction Algorithms. J. Eng. Gas. Turbines Power.

[15] Zhang M., Zhang J., Hou A., Xia A., Tuo W., Lv Y. (2023). Aerodynamic system instability identification with sample entropy algorithm based on feature extraction. Propuls. Power Res..

[16] Liu Y., Du J., Li J., Xu Y. (2023). A stall diagnosis method based on entropy feature identification in axial compressors. Int. J. Mech. Syst. Dyn..

[17] Zhang M., Kong P., Hou A., Xia A., Tuo W., Lv Y. (2022). Identification Strategy Design with the Solution of Wavelet Singular Spectral Entropy Algorithm for the Aerodynamic System Instability. Aerospace.

[18] Liu Y., Du J., Zhao D. (2025). Advances in early warning and active control of compressor instabilities for aerospace applications. Prog. Aerosp. Sci..

[19] Meng Y., Namachchivaya N.S., Perkowski N. (2025). Almost sure asymptotic stability of parabolic SPDEs with small multiplicative noise: With application to the perturbed Moore-Greitzer model. Dyn. Syst..

[20] Vepa R. (2010). Modelling and quasilinear control of compressor surge and rotating stall vibrations. Math. Probl. Eng..

[21] Mao Z., Hu J., Zhang H., Yan W. (2015). Effects of rotating disturbance on aerodynamics stability of axial-flow compressor. J. Aerosp. Power.

[22] Zhu Y., Zhu C., Tan J., Wang Y., Tao J. (2022). Operational state assessment of wind turbine gearbox based on long short-term memory networks and fuzzy synthesis. Renew. Energy.

[23] Hipple S., Bonilla-Alvarado H., Pezzini P., Shadle L.J., Bryden K.M. (2020). Using machine learning tools to predict compressor stall. J. Energy Resour. Technol..

[24] Lian R., Deng H. The improved CNN-BiLSTM hybrid model for predicting compressor stall. Proceedings of the Global Power and Propulsion Society (GPPS).

[25] Qiu X., Chen J. (2022). Algorithm of axial compressor stall warning based on BP neural network and fuzzy logic. MATEC Web Conf..

[26] Zhang M., Zhen Y., Bai S., Nan X., Ma N., Liu R., Wen Q. (2026). Graph convolutional networks modeling for aerodynamic system stability with spatiotemporal topology fusion based on digital twin. Propuls. Power Res..

[27] Deng Y., Li J., Liu J., Peng F., Zhang H., Schoen M.P. (2025). Stall prediction model based on deep learning network in axial flow compressor. Chin. J. Aeronaut..

[28] Li W., Zhang X. (2026). A hybrid DBO-VMD and IRCMRDE approach for gearbox fault diagnosis. Proc. Inst. Mech. Eng. Part C J. Mech. Eng. Sci..

[29] Chen L., Li Y. (2026). Urban Traffic Level of Service Prediction Method Based on DBO-Transformer Multi-Source Information Fusion. Promet—Traffic Transp..

[30] Hu Y., Hu A., Li B., Peng L., Han B., Fang T. (2026). Adaptive physical-layer fingerprint extraction from 100 Mbps ethernet signals using variational mode decomposition and Hilbert-Huang transform. Digit. Signal Process..

[31] Eriksen T., Rehman N.U. (2023). Data-driven nonstationary signal decomposition approaches: A comparative analysis. Sci. Rep..

[32] Chen Q., Xu Y., Zhang Z., Guo W. (2024). Feature Recognition and Extraction of Rotating Stall Signal of Mine Contra-rotating Fan. Mech. Sci. Technol. Aerosp. Eng..

[33] Zhang M., Li H., Bai S., Nan X., Ma N., Liu R., Wen Q. (2025). Collaborative Entropy Perception Application on Compressor Stall Instability with Topological Visualization. Phys. Fluids.

[34] Xue J., Shen B. (2023). Dung beetle optimizer: A new meta-heuristic algorithm for global optimization. J. Supercomput..

